# Study Progress of Noninvasive Imaging and Radiomics for Decoding the Phenotypes and Recurrence Risk of Bladder Cancer

**DOI:** 10.3389/fonc.2021.704039

**Published:** 2021-07-15

**Authors:** Xiaopan Xu, Huanjun Wang, Yan Guo, Xi Zhang, Baojuan Li, Peng Du, Yang Liu, Hongbing Lu

**Affiliations:** ^1^ School of Biomedical Engineering, Air Force Medical University, Xi’an, China; ^2^ Department of Radiology, The First Affiliated Hospital, Sun Yat-Sen University, Guangzhou, China

**Keywords:** urinary bladder cancer, multimodal imaging, radiomics, histopathological phenotype, recurrence

## Abstract

Urinary bladder cancer (BCa) is a highly prevalent disease among aged males. Precise diagnosis of tumor phenotypes and recurrence risk is of vital importance in the clinical management of BCa. Although imaging modalities such as CT and multiparametric MRI have played an essential role in the noninvasive diagnosis and prognosis of BCa, radiomics has also shown great potential in the precise diagnosis of BCa and preoperative prediction of the recurrence risk. Radiomics-empowered image interpretation can amplify the differences in tumor heterogeneity between different phenotypes, i.e., high-grade *vs.* low-grade, early-stage vs. advanced-stage, and nonmuscle-invasive *vs.* muscle-invasive. With a multimodal radiomics strategy, the recurrence risk of BCa can be preoperatively predicted, providing critical information for the clinical decision making. We thus reviewed the rapid progress in the field of medical imaging empowered by the radiomics for decoding the phenotype and recurrence risk of BCa during the past 20 years, summarizing the entire pipeline of the radiomics strategy for the definition of BCa phenotype and recurrence risk including region of interest definition, radiomics feature extraction, tumor phenotype prediction and recurrence risk stratification. We particularly focus on current pitfalls, challenges and opportunities to promote massive clinical applications of radiomics pipeline in the near future.

## Introduction

Urinary bladder cancer (BCa) is the sixth most common malignancy and the ninth most common cause of cancer death among males worldwide (1–3). An estimated 573,278 new cases and 212,536 new deaths were reported to occur in 2020 globally (3, 4). BCa is more common in men than in women, and the incidence increases with age (1, 4, 5). Meanwhile, it has a high recurrence rate (5–7). Early diagnosis with personalized treatment and follow-up of patients is critical to a favorable outcome.

BCa usually originates from the epithelium (5, 7). As carcinomas invade the detrusor muscle, they are categorized as muscle-invasive BCa (MIBC, stage ≥ T2) and more likely to metastasize to lymph nodes or other organs (5, 6). Approximately 75% of the patients at initial diagnosis have nonmuscle-invasive BCa (NMIBC, stage ≤ T1), and the rest have MIBC (6, 8–10). Nearly 50% of newly diagnosed NMIBCs are low grade, while most MIBCs are high grade (7, 11). According to the European Association of Urology (EAU) guidelines (10, 12), pathological phenotypes such as grade, stage and muscle-invasive status (MIS) are important predictors of BCa recurrence, and have immense implications for treatment decisions and prognosis. Preoperatively determining the histopathological phenotype and recurrence risk of BCa is, therefore, of critical importance for BCa patients.

The clinical first-line reference for the preoperative diagnosis of the histopathological phenotype of BCa is cystoscopic resection of a suspicious lesion during a biopsy (6, 8–10, 13, 14). Considering that bladder tumors are heterogeneous, local biopsy results may not be typical representatives of the entire tumor mass, and diagnostic errors are inevitable (5, 7, 15–19). Many studies have shown that 9 to 49% of BCa patients have their tumor stage misdiagnosed (14, 20–23), which leads to inappropriate treatment decision and unfavorable prognosis. Repeated cystoscopic resections are considered a practical way to reduce the misdiagnostic rate, but are unwanted due to the invasive, uncomfortable, time-consuming and costly process (21, 24–27). Besides, they may easily cause infection or urethral bleeding (6, 8–10, 28–30). Developing a noninvasive approach for the precise prediction of the histopathological phenotype of BCa and further stratifying its recurrence risk preoperatively is, therefore, crucial for patient treatment and management (16, 31–35).

**Figure 1:**
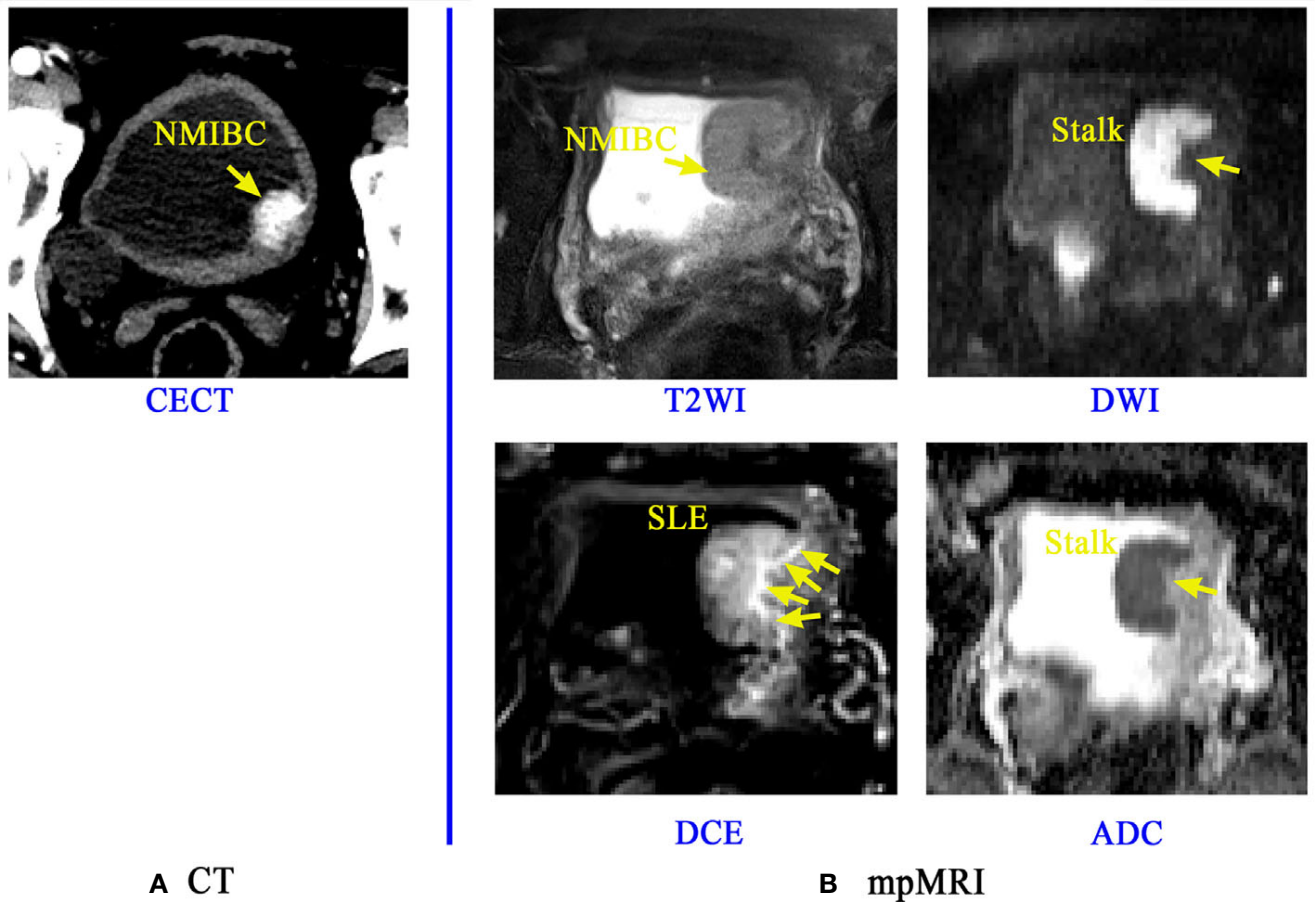
Application of CT and mpMRI for the preoperative prediction of the muscle invasion status of BCa. A lesion of a patient confirmed with NMIBC is discernible on Contrast-enhanced CT (CECT) image **(A)**, but the boundaries and basal part of this lesion is rarely distinguishable. The mpMRI **(B)** including the T2WI, DCE, DWI and its corresponding ADC map can provide more important signs and information like the stalk at the tumor base and submucosal linear enhancement (SLE) for accurate diagnosis of muscle-invasive status (MIS) of BCa (38).

In current clinical practice, easily accessible and noninvasive imaging tools such as pelvic CT and multiparametric MRI (mpMRI) provide immense assistance to clinicians for the preoperative diagnosis of BCa phenotypes (24, 30, 36–43). CT is mainly performed for evaluating the upper urinary tract and predicting lymph node metastasis of BCa (40, 42, 43). When clinicians identify the MIS, CT has drawbacks due to its limited soft-tissue contrast (40, 42, 43). In addition, radiation exposure is another concern (40, 42–44). The mpMRI, including conventional sequences like T2-weighted imaging (T2WI) and functional sequences such as diffusion-weighted imaging (DWI) with corresponding apparent diffusion coefficient (ADC) maps and dynamic contrast-enhanced imaging (DCE), may well overcome these drawbacks and enhance the diagnostic performance (**Figure 1**) (30, 39, 40, 44).

T2WI has the capability to illustrate the detailed structural information of the lesion and bladder wall, thus can potentially reflect the invasion depth of BCa into bladder wall. However, it may result in overstaging since tumor-associated inflammation has the same appearance of low signal intensity as that of the muscularis propria (20, 37, 40, 44). DWI and ADC have the favorable capability to reflect the signal intensity differences among muscle, peritumoral inflammation and fibrosis (36, 38, 44–47). The finding of a thickened hypointense submucosa beneath the NMIBC (inchworm sign or stalk) on DWI is a milestone for MIS identification and prognosis (13, 30, 41, 48). Submucosal linear enhancement (SLE) at the basal part of the tumor on DCE images has currently been recognized as another sign for precisely determining MIS (13, 30, 38, 39, 47), but its diagnostic performance is controversial (47, 49, 50).

Summarizing all these important clinical findings, Panebianco et al. proposed a Vesical Imaging-Reporting and Data System (VI-RADS), which uses tumor morphological signs, stalks and SLE on mpMRI to obtain a five-point rating score for the estimation of MIS (30, 39, 40, 51–53). However, it is a semiquantitative score which also relies most on experienced radiologists’ visual perception, making it an expert-dependent tool for BCa diagnosis. In addition, the VI-RADS model, together with the existing noninvasive imaging tools, is still incapable of predicting BCa recurrence.

During the past 20 years, the field of computer-assisted medical image analysis has grown dramatically, resulting in many successful applications in the noninvasively accurate diagnosis and prognostication of cancers such as breast cancer, colorectal cancer and lung cancer (54–57). These advances have prompted the attempt of extracting high-throughput quantitative image features, namely, *radiomics*, to characterize different tissue properties and to accumulate certain strategies for BCa phenotypes diagnosis and recurrence risk prediction (24, 26, 58–61). However, most of these radiomics strategies only focus on the tumor region, regardless of the normal wall region and the basal part of tumor region that may also provide abundant information for this task (57, 59, 60, 62). Automated and accurate delineation of regions of interest (ROI) including the tumor, its basal part and the normal wall region is an essential step toward radiomics-based bladder cancer diagnosis and prognosis. With the increasing development of radiomics, systematic analyses of these multiple regions on noninvasive bladder images would allow for a better understanding of the disease and support more personalized treatment approaches. Therefore, this review aims to extensively discuss CT- and MRI-based imaging tools and radiomics in decoding BCa phenotypes and recurrence risk, inspiring methodological progression and broadening their clinical applications in the near future.

## Search Criteria

In this study, we systematically retrieved peer-reviewed papers published from 2000 to 2021 (last query 04-20-2020). If a study appears in multiple publications, only the latest version was analyzed. The querying terms we used with the PubMed database were as:

(((((((((((((((bladder cancer[Title/Abstract]) OR (bladder tumor[Title/Abstract])) AND (CT[Title/Abstract])) OR (MRI[Title/Abstract])) OR (multiparametric MRI[Title/Abstract])) OR (radiomics[Title/Abstract])) OR (biomarker[Title/Abstract])) OR (exosome[Title/Abstract])) OR (VI-RADS[Title/Abstract])) OR (radiomics[Title/Abstract])) AND (grade[Title/Abstract])) OR (grading[Title/Abstract])) OR (stage[Title/Abstract])) OR (staging[Title/Abstract])) OR (muscle invasive bladder cancer[Title/Abstract])) OR (recurrence[Title/Abstract]).

We excluded the papers according to the following criteria: i) studies focused on nonhuman subjects; ii) studies intended to repeatedly validate the previous developed tools or important findings; iii) studies published in conference proceedings or paper responses. For each paper enrolled, the publication year, study aims, patient cohorts, methodologies, findings and limitations were specifically analyzed to extract the valuable information we need to outline the main topic of study progress on noninvasive imaging and radiomics for decoding the phenotype and recurrence risk of BCa.

## Overall Workflow

**Figure 2:**
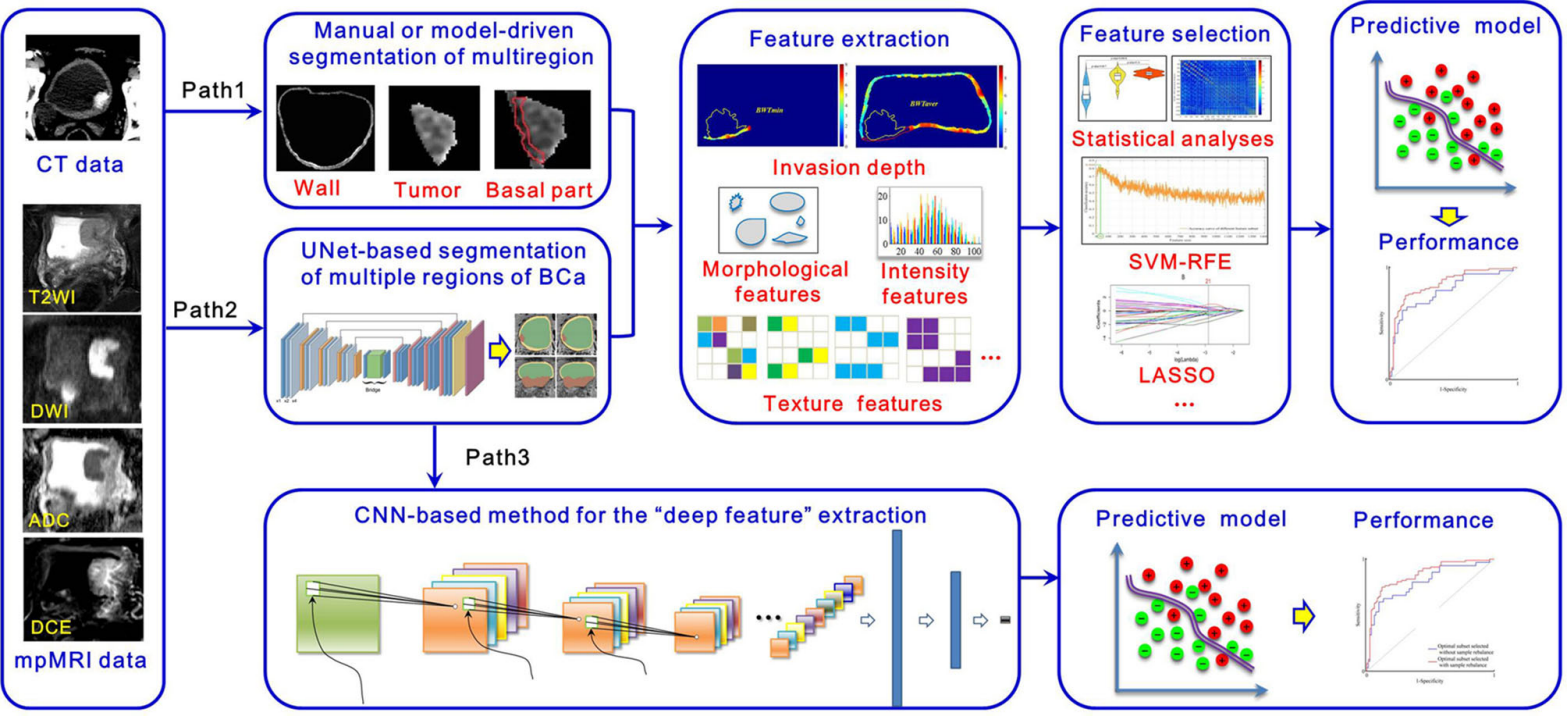
Overall workflow of the radiomics strategy for decoding BCa phenotype and recurrence risk.

According to previous studies, the overall workflow of noninvasively decoding the BCa phenotypes and recurrence risk is illustrated in **Figure 2**. Currently, the widely used imaging tools for BCa diagnosis mainly include CT, contrast-enhanced CT (CECT) and mpMRI (42, 51, 52), from which important imaging signs, such as tumor intensity distribution inhomogeneity, stalk, and SLE, can be observed by radiologists for image interpretation. After that, two radiomics pipelines, namely *Path1* and *Path 2* in **Figure 2**, are widely used to extract the high-throughput features that well reflect tumor properties for BCa phenotype prediction and recurrence risk assessment (59, 60, 62).

Apparent differences between these two pipelines are the strategies for multiregion ROIs segmentation, including the tumor region, its basal part and the normal wall region. Manual segmentation of multiregion ROIs of BCa is the first choice to many researchers. However, it is a tedious process with a huge workload. Exploring the automatic segmentation methods based on specific mathematical theorems (model-driven methods), such as level sets and Markov random fields (MRFs), becomes a more practical way. Nevertheless, owing to the intrinsic mathematical limitations, most of these methods just focus on the accurate segmentation of inner border (IB) and outer border (OB) of the bladder, incapable of segmenting the bladder multiregion on images. Consequently, some people turn to adopt the data-driven strategies like the modified UNet frame with convolutional neural network (CNN) module in *Path 2* to deal with this issue.

After image segmentation, feature extraction is the next important step. Currently, three kinds of radiomics features are commonly used, including morphological features, intensity-based features and texture features (59, 63–72). In addition, other features, such as the invasion depth of the BCa, which quantitatively measures the relative invasive depth of the tumor into the bladder wall (73), have also been gradually developed. Given that redundancy among features might severely affect the predictive performance, feature selection is indispensable toward developing an optimal predictive mode. Statistical analyses in combination with other high-level selection strategies, such as support vector machine (SVM)-based recursive feature elimination (SVM-RFE), least absolute shrinkage and selection operator (LASSO), max-relevance and min-redundancy (mRMR), are widely used (26, 61, 74, 75). With the features selected, many machine learning classifiers, such as SVM, random forest (RF), and logistic regression, can be used for prediction model development (24, 58, 74–76). These steps in *Paths 1* and *2* constitute the traditional radiomics pipelines for noninvasive prediction of BCa phenotype and recurrence risk.

Considering the rapid development of deep learning (DL) methods in disease definition and identification, we also illustrate new radiomics pipeline in *Path 3* for this task. It includes two main steps, including *i)* a segmentation step that automatically segments multiregion ROIs of BCa from the original images by using a specific CNN module and *ii)* a diagnostic step that calculates deep features from these multiregion ROIs to develop a classifier for diagnosis by using another CNN module. Owing to the “black box” nature and complex procedures used in model building, this pipeline has yet to be comprehensively investigated. With the advent of explainable artificial intelligence (AI), we believe that *Path 3* will receive much more attention and investigation in the future.

## Multiregion ROIs Extraction

**Figure 3:**
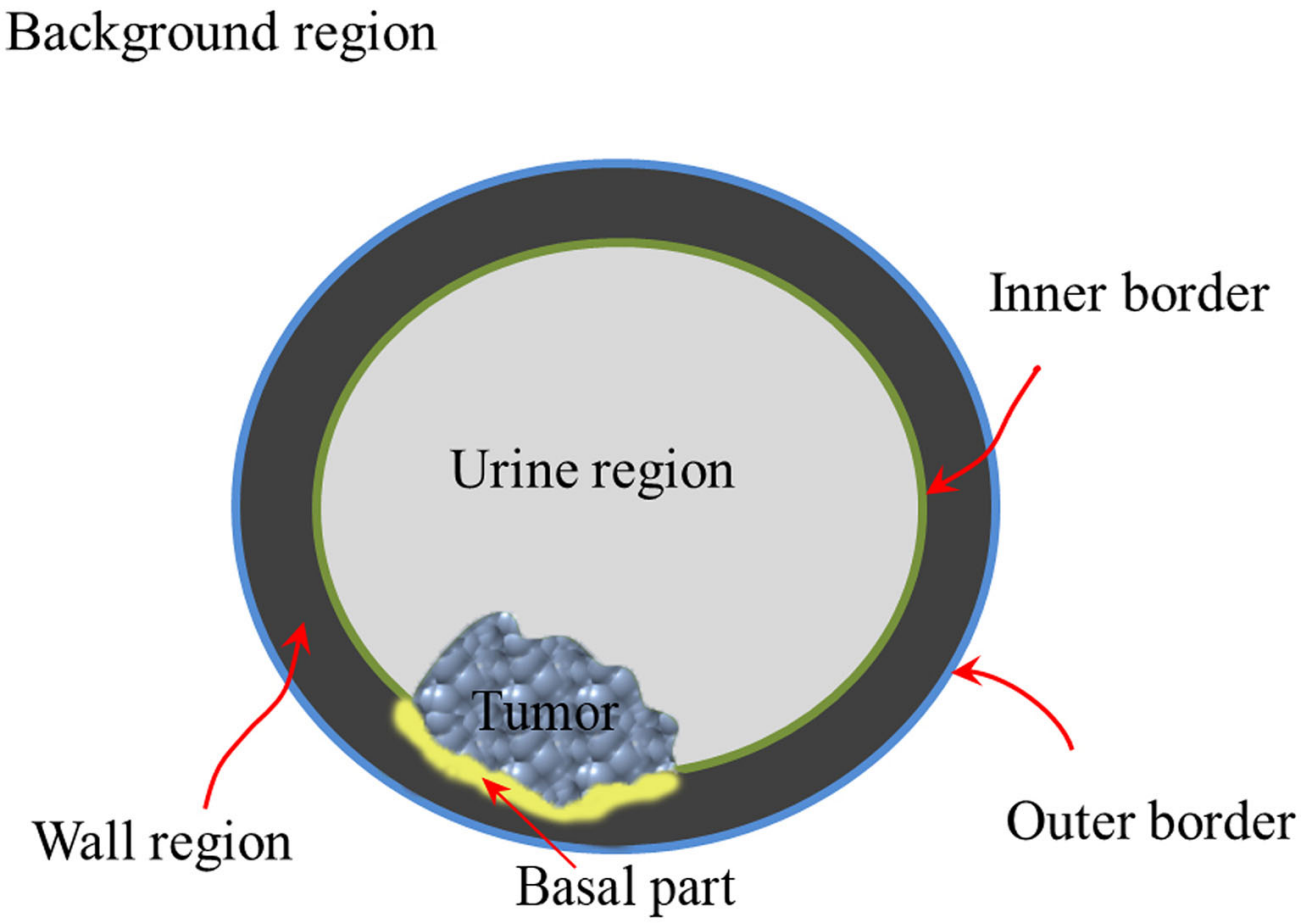
Structure diagram of the multiregion of bladder on the noninvasive image.

According to previous studies (77–82), the bladder wall and tumor regions contain plenty of information for BCa diagnosis and prognosis. A recent study (74) indicated that the basal part of bladder tumors on MRI has potential in determining MIS (**Figure 3**). Therefore, accurate delineation of the multiregion ROIs on bladder images other than using manual annotation is an essential step toward radiomics-based BCa diagnosis (83, 84).

Precise segmentation of bladder images is full of challenges, including partial volume effects, which usually occur where multiple tissues contribute to a single pixel in the image and cause blurry tissue boundaries, bladder shape variation, motion artifacts in the urine region and bladder wall, and complicated outer wall intensity distributions (83, 84). When further considering the precise segmentation of tumors in the bladder lumen, the problem becomes even more complicated (83). To address these challenges, many algorithms have been proposed since 2004 (83, 85, 86), as shown in **Table 1**. Li et al. (85, 86) first adopted the Markov random field to extract the IB of the bladder and to reduce the partial volume effects. Garnier et al. (87) adopted an active region growing strategy in a deformable model to realize the segmentation of both the IB and the OB. However, its performance for OB segmentation is far from satisfactory due to the complex tissue distribution surrounding the bladder (83).

**Table 1:** Related studies and methodology of CT-/MRI-based bladder image segmentation during the past 20 years.

Study	Imaging	Approach or strategy	Region focused	Performance and Merits
Li et al., 2004 (86)	Multispectral MRI	Partial volume (PV) scheme	IB	More information extracted from the multispectral images, and feasible for the IB.
Li et al., 2008 (85)	Multispectral MRI	Markov random field (MRF)	IB	Realizing the inhomogeneity correction and overcoming the influence of partial volume and bias field.
Duan et al., 2010 (80)	T1WI	Coupled level-sets	*IB/OB	Realizing the simultaneous extraction of both IB and OB of the bladder.
Garnier et al., 2011 (87)	T2WI	3D deformable model based on active region growing strategy	IB/OB	Achieving good performance for the IB segmentation when tumors were not existed in the bladder lumen.
Duan et al., 2011 (78)	T1WI	Coupled level-sets + volume-based features	Tumor	Realizing the automatic detection of BCa.
Duan et al., 2012 (79)	T1WI	Coupled level-sets + volume-based features + Adaptive window-setting scheme	Tumor	Realizing the automatic detection and extraction of BCa.
Ma et al., 2011 (88)	T2WI	Geodesic active contour (GAC) + shape-guided Chan-Vese	IB/OB	Achieving good segmentation performance for both bladder borders without tumor regions using two datasets with 2D images.
Han et al., 2013 (89)	T1WI	Adaptive MRF with coupled level-set constraints	IB/OB	Fast convergence, robustness to initial estimates, and robustness against noise contaminations, as well as local shape variations of the bladder wall.
Qin et al., 2014 (77)	T2WI	Coupled directional level-sets with adaptive shape prior constraints	IB/OB	With the average DSC of 0.96 and 0.946, respectively, for the IB and OB segmentation using 11 datasets.
Cha et al., 2014 (90)	#CECT	Conjoint level set analysis and segmentation system (CLASS)	IB/OB	With the average DSC of 0.842 for the IB segmentation using 182 datasets.
Dolz et al., 2018 (83)	T2WI	Progressive dilated convolution-based U-NET model	IB/OB/Tumor	With the average DSC of 0.9836, 0.8391 and 0.6856, respectively, for the IB, OB and tumor region segmentation using 60 datasets.
Gordon et al., *2018* (91)	CECT	Deep-learning convolutional neural network (DL-CNN)	IB/OB	With the average DSC of 0.9869 and 0.875, respectively, for the IB and OB segmentation using 172 datasets.
Ma et al., 2019 (92)	CECT	U-Net–based deep learning approach (U-DL)	IB	With the average DSC of 0.934 for the IB segmentation using 173 datasets.

*IB and OB represent the inner and outer borders of bladder, respectively.

^#^CECT indicates contrast-enhanced CT.

Almost at the same time, level-set-based methods were introduced to extract both the IB and OB (77, 79, 80, 88, 89, 93). Duan et al. (80, 93) first proposed a coupled level-set framework with the modified Chan–Vese model to locate IB and OB from T1-weighted imaging (T1WI) in a 2-dimensional (2D) slice fashion. Based on the merits of this method for IB segmentation, Duan et al. (78, 79) further proposed an adaptive window-setting scheme with volume-based features to extract tumors on IB. Shortly afterward, Ma et al. (88) introduced the geodesic active contour (GAC) scheme into the Chan-Vese model to realize the shape-guided deformation of both IB and OB on the T2WI. A limitation of this approach is the intensity bias induced by the tumors inside the bladder lumen that easily leads to the leakage of IB segmentation. To overcome this limitation, Qin et al. (77) proposed an adaptive shape prior constrained level-set algorithm that evolves both IB and OB simultaneously from T2WI, greatly improving the accuracy for IB and OB segmentation. However, level-set-based methods are modality-dependent and cannot be freely applied among different sequences or modalities. In addition, none of these methods can realize the simultaneous location and evolution of IB, OB and tumor regions.

Recently, CNN-based DL strategies have emerged as powerful tools for the semantic segmentation of bladder lumen CT images (90–92). During 2018, our group (83) proposed a modified UNet framework with a progressive dilated CNN module, realizing the simultaneous segmentation of IB, OB and BCa on T2WI for the first time. The average Dice’s coefficient (DSC) of IB and OB were 0.9836 and 0.8391, respectively, but that of the tumor region was only 0.6856 (83).

**Figure 4:**
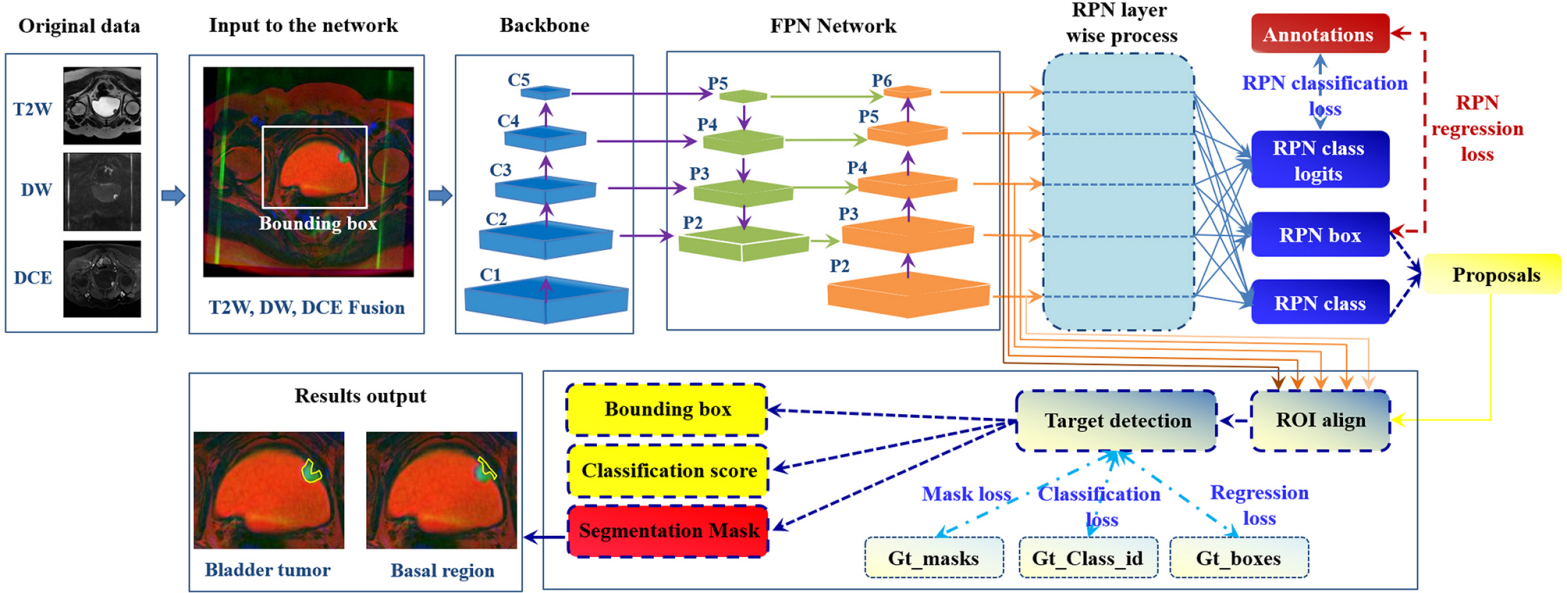
Future framework of simultaneous segmentation of the multi-target regions from the bladder mpMRI. The Gt_class_id, Gt_boxes, and Gt_masks represent the ground truth of the multiregion anatation, position of the regions to be detected and focused, and segmentation mask (94).

Considering that different imaging sequences could provide complementary information for BCa diagnosis, how to realize the simultaneous segmentation of the multiple target regions on mpMRI bladder images becomes the ultimate goal in the workflow (**Figure 1**). To this end, we design an automatic bladder multiregion segmentation framework in **Figure 4**, which is based on the Mask-R-CNN (94) and mpMRI fusion strategy (95) with multiple labels to realize multiregion segmentation of mpMRI bladder images.

## Radiomics-Empowered Diagnosis of BCa Phenotype

### BCa Grading

The histological grade of BCa is a critical factor for the treatment decisions and prognosis (96). Cystoscopic resection and biopsy remains standard reference for BCa grading (76), but may easily cause diagnostic error due to the heterogeniety of tumor tissues (76).

With the development of noninvasive imaging, the imaging signs that reflect the BCa grade have been successively unearthed (96–102). For example, the peak time enhancement in the first minute (E_max/1_) after contrast administration and the steepest slope of the DCE were first reported to be closely related to tumor angiogenesis (97). ADC values, including the mean ADC value and the normalized ADC value derived from DWI, have been demonstrated to be useful for BCa grading (98–103). In particular, Rosenkrantz et al. (37) adopted the quantitative metrics extracted from the tumor region on T2WI and DWI, including the tumor diameter, normalized T2 signal intensity and mean ADC value, for the assessment of tumor grade, as shown in **Table 2**. Although statistical analysis indicated that only the mean ADC value was a significant predictor, an area under the curve (AUC) of 0.804 was achieved for BCa grading (37), which could be recognized as the embryonic form of the mpMRI radiomics concept for BCa diagnosis.

**Table 2:** Related studies and strategies of CT-/MRI-based BCa grading during the past 20 years.

Study	Patient	Imaging		Target	Approach or strategy	Results and findings
Tuncbilek et al., 2009 (97)	24 patients from single center	DCE		Tumor	Extracting *peak time* *enhancement in the first* (E_max/1_), *second* (E_max/2_), *third* (E_max/3_), *fourth* (E_max/4_) and *fifth* (E_max/5_) minute after contrast administration, and the *steepest slope for* statistical analysis with tumor grade.	E_max/1_and *steepest slope* had statistically significant correlation with tumor grade.
Avcu et al., 2011 (98)	63 patients from single center	DWI		Tumor	*Mean ADC values* were measured from the tumor mass.	The *mean ADC value* were significantly different between the high- and low-grade BCa.
Rosenkrantz et al., 2013 (37)	37 patients from double centers	T2WI, DWI		Tumor	*Tumor diameter, normalized T2 signal intensity* and *mean ADC value* were extracted.	*Mean ADC value* was statistically significant between the high- and low-grade BCa, with an AUC of 0.804 for the classification of this two groups.
Kobayashi et al., 2014 (104)	132 patients from single center	DWI		Tumor	*Mean ADC value* was calculated.	*Mean ADC value* was significantly lower in tumors with higher Ki-67 Lis and higher grade.
Sevcenco et al., 2014 (105)	43 patients from single center	DWI		Tumor	*Mean ADC value* was obtained.	*Mean ADC value* achieved favorable performance in predicting tumor grade, with an AUC of 0.906.
Sevcenco et al., 2014 (106)	41 patients from single center	DWI		Tumor	*Mean ADC value, p53 and p21* were obtained.	*Mean ADC value* and *p21* were the independent predictors for BCa grade, with an AUC of 0.981.
Wang et al., 2014 (102)	30 patients from single center	DWI		Tumor and referenced regions like urine	*Mean ADC value* and *normalized ADC (nADC)* values were calculated.	The performance of using the *nADC* with urine as reference was the best, with the AUC of 0.995.
Zhang et al., 2017 (107)	128 patients from single center	*CECT		Tumor	Six texture features, including *mean*, *SD*, *entropy*, *mean of positive pixels (MPP)*, *skewness* and *kurtosis*, were extracted.	*Mean*, *entropy* and *MPP* were significantly different between the high-grade BCa and low-grade on both unenhanced and enhanced images. *MPP* obtained from unenhanced images achieved the best performacne, with the AUC of 0.779.
Mammen et al., 2017 (108)	48 patients from single center	CT		Tumor	Texture features including *Kurtosis*, *skewness* and *entropy*, were extracted.	Only entropy showed significant inter-group differences, and it achieved an AUC of 0.83 in differentiation of low- and high-grade BCa.
Zhang et al., 2017 (25)	61 patients form single center	DWI ADC maps		Tumor	102 radiomics features, including the histogram and GLCM features	The model developed could achieve favorable performance for BCa grading, with the AUC of 0.861, significantly better than that of using the ADC value alone.
Wang et al., 2019 (76)	100 patients from single center	T2WI, DWI and ADC maps		Tumor	924 features were extracted, including morphological features and six categories of texture features like histogram features, GLCM features, *GLRLM features, *GLSZM features, *NGTDM features, and *GLDM features.	The multi-modal MRI-based radiomics approach has the potential in preoperative grading of BCa, with the AUC of 0.9276.
Wang et al., 2020 (15)	58 patients from single center	T2*-weighted imaging and DWI		Tumor	Apparent transverse relaxation rate R2* and *mean ADC value* were calculated.	R2* and *mean ADC value* were significantly different between low- and high-grade BCa, with the AUC of 0.714 and 0.779 in the classification process, respectively.
Zhang et al., 2020 (109)	145 patients from single center	CT	Tumor		1316 radiomics features, involving the morphological features, histogram features, GLCM features, GLRLM features, GLSZM features, GLDM features, were calculated.	The proposed radiomics model achieved a good performance, with AUC of 0.85 using the testing cohort.

*CECT indicates the contrast enhanced CT.

*GLRLM indicates the gray-level run length matrix; GLSZM indicates the gray-level size zone matrix; NGTDM indicates the neighborhood gray tone difference matrix; GLDM indicates the gray-level dependence matrix.

In 2017, our group proposed a radiomics framework and investigated its feasibility for BCa grading (25). We adopted 102 radiomics features involving the histogram features and gray-level co-occurrence matrix-based (GLCM) features from the DWI and ADC maps to quantitatively describe the tumor properties. Then, the Mann–Whitney U-test and SVM-RFE were adopted for feature selection and diagnostic model development. The results based on 61 patients showed that the diagnostic model achieved a favorable performance for BCa grading, with an AUC of 0.861, which was significantly better than that of using the mean ADC values alone. Afterward, Wang et al. (76) investigated the performance of using the radiomics strategy with T2WI, DWI and ADC maps for BCa grading, achieving a more favorable diagnostic performance with an AUC of 0.9276 (76).

In addition, several studies have attempted to extract texture features from the tumor region on CT images for BCa grading. First-order texture features, such as the mean, standard deviation (SD), entropy, mean of positive pixels (MPP), skewness and kurtosis, and second-order features, such as GLCM features and gray-level run-length matrix (GLRLM) features, are commonly used and achieved the highest AUC of 0.83 (107–109).

### MIS Prediction and Staging

Accurately predicting the stage and MIS of BCa is also crucial in making treatment decisions (37, 47, 105, 106). Pathological examination of transurethral resection of bladder tumor (TURBT) specimens is the first-line reference for preoperative BCa staging (38, 44, 47, 49, 51, 110). However, it may cause diagnostic errors such as understaging, misleading clinicians in making decisions (38, 44, 47, 51, 110, 111). A previous study reported that the error rate for preoperative BCa staging varies from 20 to 80% (20).

In current clinical practice, noninvasive imaging tools such as CT and MRI are also widely used for BCa staging and MIS prediction (15, 49, 51, 52, 112). However, the precision and robustness of using these imaging tools are unsatisfactory due to the challenges of discriminating between submucosal invasion and muscle invasion and between muscle invasion and perivesical fat proliferation by visual perception (15, 47, 50, 51, 112).

During 2000, Hayashi et al. (49) observed that the image sign of SLE often appears on NMIBC patients’ DCE images (50). This finding is undoubtedly a milestone in imaging-based diagnosis of BCa stage and MIS. Afterward, Takeuchi et al. (44, 50) reported another important sign named the submucosal stalk or “inchworm” sign found among most NMIBCs on DWI, fortifying the precision and robustness of imaging-based diagnosis of BCa stage and MIS (49). Then, many studies found that the ADC values derived from high-stage (≥ T2) bladder tumors on DWI were significantly lower than those from low-stage (≤ T1) bladder tumors and thus could be used for the quantitative diagnosis of BCa stage and MIS with AUCs roughly between 0.65 and 0.96 (37, 38, 47, 49, 52, 104, 105, 110), as shown in **Table 3**.

**Table 3:** Related studies and strategies of CT-/MRI-based BCa staging and MIS prediction during the past 20 years.

Study	Patient	Imaging	Target	Approach or strategy	Results and findings
Hayashi et al., 2000 (49)	71 patients from single center	DCE	Tumor	*Submucosal linear enhancement* (SLE)	*SLE* achieved an accuracy of 83% for BCa staging, and 87% for MIS prediction, respectively.
Takeuchi et al., 2009 (41)	40 patients with 52 bladder tumors from single center	T2WI, DWI, DCE	Tumor	*Submucosal stalk*	The overall accuracy of T stage diagnosis was 67% for T2WI alone, 88% for T2WI+ DWI, 79% for T2WI+DCE, and 92% for all three image types together.
Rosenkrantz et al., 2013 (37)	37 patients from double centers	T2WI, DWI	Tumor	*Tumor diameter, normalized T2 signal intensity* and *mean ADC value* were extracted.	High-stage (≥ T2) tumors showed greater tumor diameter and lower mean ADC value than the low-stage (≤ T1) tumors. The AUC for MIS prediction was 0.804 by jointly using the tumor diameter and mean ADC value.
Kobayashi et al., 2014 (104)	132 patients from single center	DWI	Tumor	*Mean ADC value* was calculated.	*Mean ADC value* was significantly lower with higher T stage bladder tumors.
Sevcenco et al., 2014 (105)	43 patients from single center	DWI	Tumor	*Mean ADC value* was obtained.	*Mean ADC value* achieved good performance in predicting MIS, with an AUC of 0.884.
Wang et al., 2016 (38)	59 patients from single center	T2WI, DWI, DCE	Tumor	*SLE, submucosal stalk*	The staging accuracy of DWI was 91.3%. When combining with DCE, the accuracy was improved to 94.6%.
Xu et al., 2017 (24)	68 patients from a single center	T2WI	Tumor	*A total of 63 three-dimensional radiomics features, including the histogram-based features and GLCM features, were extracted from the original images and their high-order derivative maps in association with the Student’s *t*-test and SVM-RFE for feature selection and SVM classifier for the diagnostic model development.	13 features were finally selected, with an optimal AUC of 0.8610 for MIS diagnosis, which for the first time introduced the radiomics strategy into the preoperative MIS identification and demonstrated its feasibility.
Wu et al., 2017 (113)	118 patients from single center	CT	Tumor	# A radiomics signature was determined by the optimal features selected from the original 150 radiomics features uing the LASSO approach. In combination with the clinical factors, a radiomics nomogram was then developed.	The radiomics nomogram showed good discrimination in training and validation cohorts for the prediction of lymph node metastasis, with the AUC of 0.9262 and 0.8986, respectively.
Panebianco et al., 2018 (114)	/	T2WI, DWI, ADC, DCE	Tumor and submucosal layer	Quantitatively scoring the imaging signs like tumor shape, stalk and SLE on the multiparametric MRI.	The Vesical Imaging-Reporting and Data System (VI-RADS) could be a standard and useful tool to half quantify these imaging signs on the multiparametric MRI for BCa staging and MIS diagnosis.
Wu et al., 2018 (29)	103 patients from single center	T2WI	Tumor	A radiomics signature was determined by nine optimal features selected from the original 718 radiomics features uing the LASSO approach. In combination with the clinical factors, a radiomics nomogram was then developed.	The radiomics signature achieved the AUC of 0.8447 for the prediction of lymph node metastasis. And the nomogram consisted of the radiomics signature with the clinical factors achieved more favorable performance, with the AUC improved to 0.8902 in the validation cohort.
Xu et al.,2019 (26)	54 patients from single center	T2WI, DWI, ADC	Tumor	Radiomics features like histogram-based, GLCM and GLRLM features were extracted from the multimodal MRI data with the multi-grayscale normalization strategy.	The optimal 19 features derived from the three modalities finally achieved the best performance, with the AUC of 0.9756 for MIS diagnosis, indicating the great capacity of the multimodal MRI-based radiomics strategy for the preoperative MIS identification.
Zheng et al., 2019 (30)	199 patients from single center	T2WI	Tumor and basal part	2602 radiomics features were extracted from both the tumorous region and basal part of the images. A radiomics signature was determined uing the LASSO approach. In combination with the clinical factors, a radiomics nomogram was then developed.	The radiomics signature showed good performance in MIS prediction. Integrating with the clinical factor, nomogram achieved much better diagnostic power, with the AUC improved to 0.876 in the validation cohort.
Barchetti et al., 2019 (51)	78 patients from single center	T2WI, DWI, ADC, DCE	Tumor and submucosal layer	VI-RADS	The VI-RADS achieved favorable performance for MIS diagnosis, with the AUC of 0.926 and 0.873 when conducted by reader 1 and 2, respectively.
Ueno et al., 2019 (39)	74 patients from single center	T2WI, DWI, ADC, DCE	Tumor and submucosal layer	VI-RADS	The VI-RADS achieved favorable performance for MIS diagnosis, with pooled AUC of 0.90 when conducted by five readers.
Wang et al., 2019 (40)	340 patients from single center	T2WI, DWI, ADC, DCE	Tumor and submucosal layer	VI-RADS	The VI-RADS achieved excellent performance for MIS diagnosis, with the AUC of 0.94 when conducted by two readers in consensus.
Wang et al., 2020 (115)	106 patients from double centers	T2WI, DWI, ADC	Tumor	1404 radiomics features were extracted. A radiomics signature was generated using the SVM-RFE and logistic regression. A nomogram was then developed using the signature and MRI-determined tumor stalk.	The signature alone achieved a good performance in MIS prediction. The nomogram integrating with the signature and tumor stalk achieved much better diagnostic performance, with the AUC improved to 0.877 in the validation cohort.

*SVM-RFE indicates the support vector-machine-based recursive feature elimination algorithm.

^#^LASSO indicates the least absolute shrinkage and selection operator algorithm for feature selection.

By integrating all of these imaging signs, Panebianco et al. (114) proposed VI-RADS to quantify these signs on mpMRI and further standardize the image-based diagnostic procedures for MIS prediction (44, 45, 114). The performance was then evaluated by three groups, with the AUC varying between 0.873 and 0.94 (39, 40, 51, 111). Although VI-RADS has integrated all of the existing imaging signs, such as tumor intensity inhomogeneity, stalk and SLE, into the scoring system for MIS prediction, it is still a semiqualitative and expert-dependent process. Radiomics models based on high-throughput quantitative image features to implement automatic prediction of tumor phenotypes are considered a more practical method.

In fact, before VI-RADS was proposed, we reported the first radiomics strategy for the MIS prediction of BCa (24). This strategy utilized 63 radiomics features, including the histogram-based features and GLCM features extracted from the original T2WI and its high-order derivative maps for tumor characterization, achieving an AUC of 0.861 in MIS prediction (24). Shortly afterward, we extracted the GLCM and GLRLM features from the T2WI, DWI and ADC images and achieved a great performance improvement in MIS prediction, with an AUC of 0.9756 (26). Then, Zhang et al. (30) creatively included both the tumor region and the basal part with a radiomics nomogram that was proposed by Wu (29, 113), indicating that the basal part of bladder tumors is also critical for BCa MIS prediction.

All of these radiomics-based studies were based on single-center data. In 2020, we collected a double-centered mpMRI database involving 106 eligible patients, and adopted five categories of texture features and clinical factors to develop a new nomogram model for MIS prediction, achieving AUCs of 0.924 and 0.877 in both the training and validation cohorts, respectively (115).

## Radiomics-Empowered Stratification of BCa Recurrence Risk

A high recurrence rate is a distinguishing epidemiological property of BCa. The recurrence rate of NMIBC patients who underwent TURBT at one year was as high as 70% (8, 10, 112). However, as many as 50% of MIBC patients who undergo radical cystectomy (RC) with bilateral lymph node dissection and ileal conduits develop local or metastatic recurrence during the next 24 months (61, 116, 117). Preoperatively predicting the recurrence risk of BCa patients is pivotal for facilitating appropriate adjuvant treatment strategies and the management of patients.

**Figure 5:**
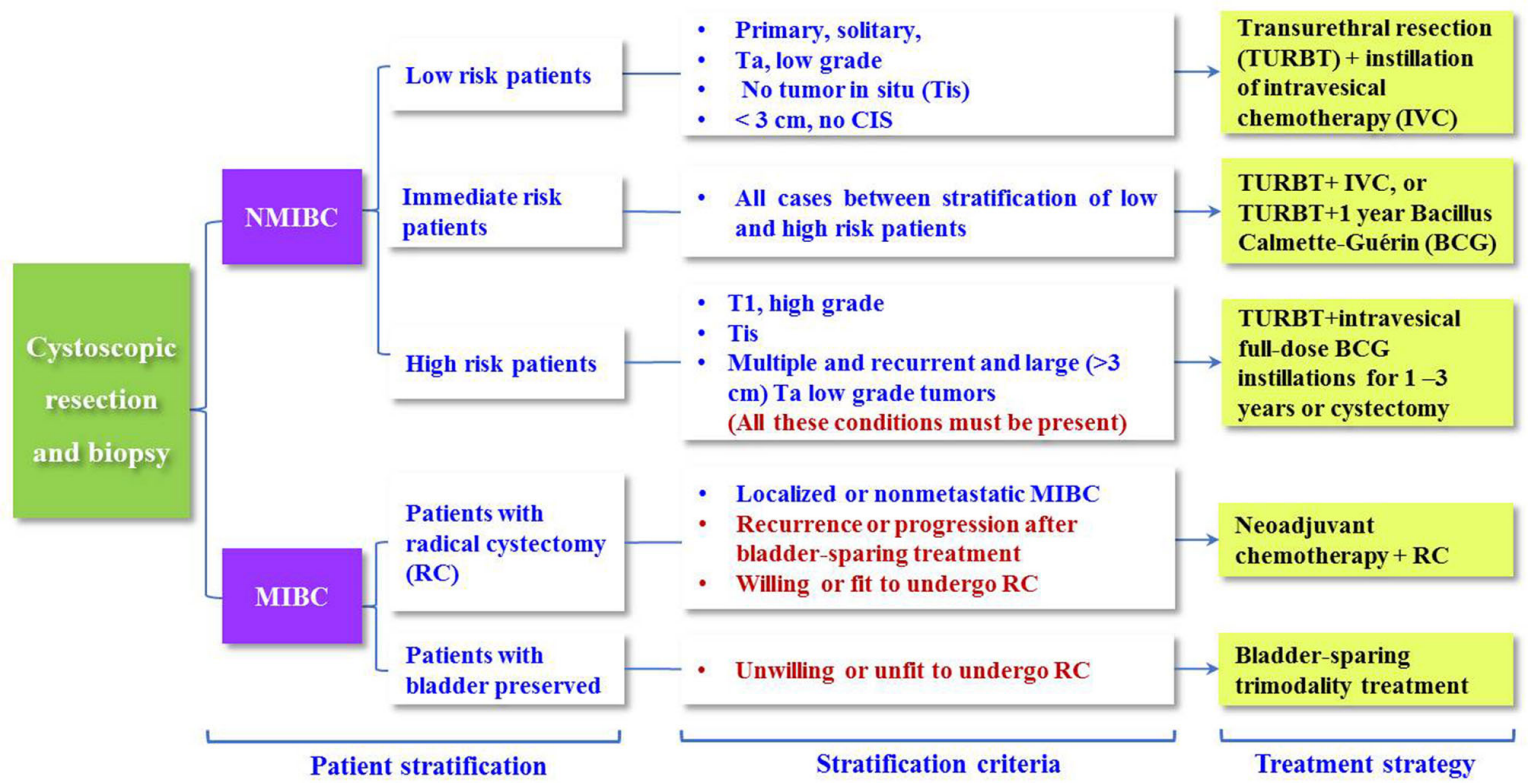
Treatment recommendations for BCa patients based on the MIS, grade and recurrence risk stratification.

At present, the EAU has provided guidelines to stratify BCa patients into different groups to recommend more specific adjuvant therapy (8, 10, 15, 29, 112), as shown in **Figure 5**. The guidelines categorize NMIBC patients into low-, intermediate- and high-risk groups of recurrence using the European Organization for the Research and Treatment of Cancer (EORTC) risk table and recommend TURBT + intravesical chemotherapy (IVC), TURBT + one-year Bacillus Calmette-Guérin (BCG), and RC. Nevertheless, this risk table merely considers six predominant clinical and histopathological factors, including the number of tumors, tumor size, prior recurrence rate, T stage, grade, and presence of concurrent tumors *in situ* (Tis), to achieve a quantitative prediction of the recurrence risk (10, 29).

Then, the Club Urológico Español de Tratamiento Oncológico (CUETO) developed a new risk table to predict the short- and long-term recurrence risks for NMIBC patients with postoperative BCG treatment (15). Many studies subsequently reported that the precision of the EORTC and CUETO risk tables was far less than satisfactory in the recurrence risk stratification of NMIBC, with Harrell’s C-index ranging between 0.51 and 0.77 (8, 10, 35, 48, 118–122), as shown in **Table 4**. Other studies also reported that tumor sites in the bladder neck and/or trigone, grade and stage are independent risk factors for the prediction of BCa recurrence (48, 117, 123). In 2019, Yajima et al. (48) found that the tumor stalk (inchworm sign) on DWI is a significant sign for BCa prognosis.

**Table 4:** Related studies and strategies of BCa recurrence risk prediction during the past 20 years.

Study	Patient	Treatment	Follow-up/years	Predictionmodel	Findings	Conclusion
Sylvester et al., 2006 (118)	2596 NMIBC patients from 7 EORTC trials	TURBT + Intravesical treatment (78.4% of the patients)	Median follow-up of 3.9 years and maximum follow-up of 14.8 years	Univariate and multivariate analyses	The EORTC risk table was derived based on the number and size of tumors, prior recurrence rate, T category, carcinoma in situ, and grade.	EORTC risk table is a useful tool for the urologist to discuss the different options with the patient to determine the most appropriate treatment and frequency of follow-up.
Fernandez et al., 2009 (8)	1062 NMIBC patients from 4 CUETO trials	TURBT + BCG with 12 instillations	5 years	Univariate and multivariate analyses	The CUETO risk table was developed using gender, age, grade, tumor status, multiplicity and associated Tis.	The recurrence risks calculated by the CUETO table were lower than those obtained with EROTC table.
Seo et al., 2010 (122)	251 patients from single center	TURBT + full-doze maintenance BCG	5 years and 9 months	EORTC	C-index: 0.62	The recurrence rate and progression rate were almost similar to the EORTC risk tables. However, the recurrence rate was low in the intermediate-risk group.
Xylinas et al., 2013 (120)	4784 patients from 8 centers	TURBT +51% cohort of immediate single postoperative chemotherapy + 11% cohort of BCG	4 years and 9 months	EORTC, CUETO	C-index: 0.60, 0.52	Both models exhibited poor discrimination. Specific biomarkers should be exploited for improving the performance.
Xu et al., 2013 (48)	363 NMIBC patients from single center	TURBT +79% cohort of immediate single postoperative chemotherapy + 100% cohort of the entire course of intravesical chemotherapy	3 years	EORTC, CUETO	C-Index: 0.71, 0.66	The EORTC model showed more value in predicting recurrence and progression in patients with NMIBC.
Kohjimoto et al., 2014 (121)	366 NMIBC patients from single center	TURBT + BCG	5 years	EORTC, CUETO	C-index: 0.51, 0.58	Although both exhibited poorly for recurrence prediction, CUETO was a little better.
Vedder et al., 2014 (35)	1892 NMIBC patients from 18 centers	TURBT +13~22% cohort of the entire course of intravesical chemotherapy+17~30% cohort of BCG + 0.55~0.61% cohort of Re-TURBT	10 years	EORTC, CUETO	C-index: 0.56-0.59, 0.64-0.72	The discriminatory ability for BCa recurrence was unsatisfactory.
Cambier et al., 2016 (10)	1812 NMIBC patients from 2 EORTC trials	TURBT + 1~3 years of maintenance BCG	7 years 5 months	Updated EORTC	C-index: 0.59.	NMIBC patients treated with1~3 years of maintenance BCG had a heterogeneous prognosis among the high-risk patients, and early cystoscopy should be considered.
Dalkilic et al., 2018 (119)	400 NMIBC patients from single center	TURBT + BCG (45.3% of the patients)	5 years	EORTC, CUETO	C-index: 0.777, 0.703	EORTC risk table was better than the CUETO table for the recurrence prediction.
Kim et al., 2019 (35)	970 NMIBC patients from single center	TURBT + BCG	5 years	New model, EORTC	AUC: 0.65, 0.56	The new model developed by using gross hamartia, previous or concomitant upper urinary tract urothelial carcinoma, stage, grade, number of tumors, intravesical treatment performed better than the EORTC risk table.
Yajima et al., 2019 (48)	91 NMIBC patients from single center	TURBT	5 years	Inchworm sign (tumor stalk) on the DWI and ADC images	The progression rate of inchworm-sign-negative cases was significantly higher than that of inchworm-sign-positive cases, whereas there was no significant difference in the recurrence rate between two groups.	The absence of an inchworm sign and histological grade 3 were independent risk factors for progression.
Xu et al., 2019 (61)	71 patients including 36 NMIBC patients and 35 MIBC patients from single center	TURBT for the NMIBC patients and RC for the MIBC patients	2 years	Radiomics nomogram developed based on the radiomics features extracted from T2WI, DWI, ADC, and DCE MRI data, and the clinical risk factors	The proposed radiomics nomogram exhibited good performance both in the training cohort (AUC: 0.915) and the validation cohort (AUC: 0.838) for the prediction of the BCa recurrence during 2 years after operation.	The proposed radiomics-clinical nomogram has potential in the preoperative prediction

Considering that the high-throughput radiomics features of the underlying tumor region have the potential to reflect tumor heterogeneity and the microenvironment, which are closely related to tumor recurrence, making full use of these features may achieve a more accurate prediction of the risk of BCa recurrence.

With this assumption, our group retrospectively collected the preoperative T2WI, DWI, ADC and DCE images of 71 patients who were confirmed with NMIBC or MIBC, treated with TURBT or RC accordingly, and followed for 2 years (61). Then, 1872 radiomics features were extracted from the tumor regions of their preoperative mpMRI, including histogram features, GLCM features, GLRLM features, neighborhood gray-tone difference matrix (NGTDM) features and gray-level size zone matrix (GLSZM) features. After that, these features in combination with important clinical risk factors, such as age, sex, grade, MIS, stalk, SLE, tumor size, number of lesions and surgery choice (TURBT or RC), were used for radiomics-clinical nomogram development. The performance of the nomogram model obtained AUCs of 0.915 and 0.838 for the training and validation cohorts, respectively. These results suggest that the radiomics strategy has excellent potential in the preoperative prediction of BCa recurrence.

## Discussion and Future Perspectives

Urinary bladder cancer is a highly prevalent disease among aged males (1–3). Accurate diagnosis of tumor phenotypes and recurrence risk serves as the “bedrock” of appropriate clinical therapeutic strategy and is of vital importance in the follow-up management of BCa patients. The standard reference for preoperatively diagnosing BCa phenotypes is cystoscopic biopsy, which is an invasive procedure that carries certain risks of bladder perforation (30). More importantly, a significant risk of misdiagnosis such as understaging or overstaging, may occur that induces incorrect estimation of the recurrence risk based on EORTC, and delays the proper radical treatment (8, 10, 13, 30).

In recent years, reading preoperative radiographic images produced by CT, CECT, PET, mpMRI, or US plays an essential role in the noninvasive diagnosis and recurrence prediction of BCa, in which radiomics strategies have also demonstrated their great power of identifying complex patterns precisely, effectively and stably (124). Integrating radiomics strategies with noninvasive imaging in the clinical setting is expected to provide more valuable supplementary information to the urologist for BCa diagnosis and prognosis, preoperatively.

However, the clinical application of noninvasive imaging-based radiomics strategies for preoperatively decoding BCa phenotypes and recurrence risk is still in its infancy. In this study, we reviewed the rapid progress in the field during the past 20 years, summarizing the entire pipeline of the radiomics strategy including region of interest definition, radiomics feature extraction, tumor phenotype prediction and recurrence risk stratification, sincerely hoping to further promote massive clinical applications of noninvasive radiomics tools for the preoperative BCa diagnosis and prognosis in the near future.

In this section, we particularly focused on the current pitfalls, challenges and opportunities of this field.

### Public Imaging Datasets for BCa

Data collection is the first step to adopt radiomics strategies for the BCa phenotype and recurrence risk prediction. At present, there are several public databases for BCa research, including the *National Cancer Database* (NCDB), the *National Cancer Institute’s Surveillance, Epidemiology, and End Results* cancer database (SEER) (125), and *The Cancer Imaging Archive* database (TCIA). Although the first two databases contain nearly 100 thousand BCa patients, most of them only contain the clinical diagnoses, treatments and end results, without the imaging datasets attached. TCIA aims to deidentify and host a large archive of medical images of cancer accessible for public research. However, it contains only 139 BCa patients’ medical images. Therefore, the current public datasets are very limited for developing a radiomics model with sufficient training and testing for the prediction task.

### Simultaneous Segmentation of Multiple Regions From Multimodal Bladder Images

Precise segmentation of multiple regions of the bladder on images, including tumor regions, basal parts, and bladder wall regions, is a critical step toward further extracting features for tumor phenotype prediction. Several previous studies adopted a two-step strategy to first segment the mixed region between IB and OB from the original image and then separate the tumor lesion from its adherent wall region (78, 79, 81). This strategy not only reduces the segmentation precision but also increases the complexity and time consumption.

So far, only one study implemented the simultaneous segmentation of the IB, OB and tumor regions from the bladder images (83), but its performance for tumor segmentation was unsatisfactory. As indicated in **Figure 4**, it is expected that the end-to-end framework based on the DL networks could facilitate better segmentation performance (126–129). In particular, with more domain priors, such as the bladder wall thickness distribution, shape variation and attention mechanism of the integrated target region (13, 30, 39, 53), more precise and robust DL-based models could be established to improve the accuracy and efficiency of multiregional bladder segmentation from multimodal images, such as mpMRI.

### Quantitative Invasion Depth Definition for BCa Staging

**Figure 6:**
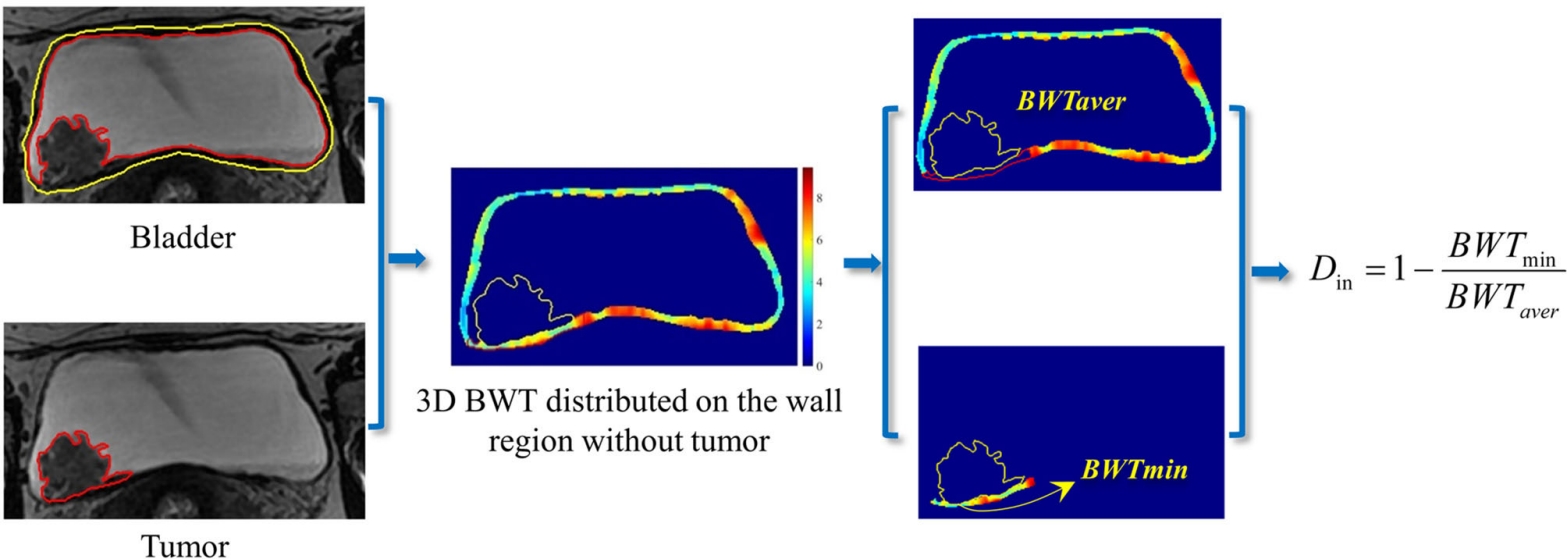
A potential definition of the invasive depth of bladder tumor based on the BWT distribution on the bladder wall region.

Almost all of the previous studies were focused on the tumor region for feature extraction (24, 107, 109, 130, 131). Currently, only one study considered both the tumor region and the basal part for radiomics feature calculation and it reported the superiority of this new strategy for staging and MIS prediction (74). Considering that the bladder wall region also contains useful information such as bladder wall thickness (BWT) for BCa detection and diagnosis (81, 132), more features are expected to be designed for BCa staging and MIS prediction. For instance, using the tumor location and BWT distributed on the wall region, the invasive depth of BCa (*D_in_*) might be defined by the entropy of minimum BWT (*BWT_min_*) of the cancerous region and the average BWT (*BWT_aver_*) other than the cancerous region, as shown in **Figure 6**.

### Fully Using VI-RADS for BCa Phenotype Prediction and Recurrence Risk Stratification

During the past 20 years, mpMRI is increasingly introduced into pre-TURBT diagnosis, achieving favorable accuracy in BCa staging and differentiation of NMIBC and MIBC (30, 39, 40). Despite the undeniable advances in mpMRI for bladder imaging, a lack of standardization of imaging protocols and reporting basis becomes the main cause of performance variation. To this end, VI-RADS scoring system defines a standardized approach to imaging and reporting mpMRI for BCa (39). Nevertheless, most of the previous studies only focused the performance of using VI-RADS for the pre-TURBT discrimination between NMIBC and MIBC (13, 30, 51, 53), regardless of other valuable diagnostic information VI-RADS may contain for therapeutic strategy (133, 134).

Del Giudice et al. (135, 136), recently reported that *i)* VI-RADS could provide valuable information for the selection of patients who are candidate for repeated-TURBT among the high-risk NMIBC cases; *ii)* VI-RADS could be valid and reliable in discriminating between BCa patients with extravesical disease and those with muscle-confined BCa before TURBT, and VI-RADS score 5 could be used to predict significant delay in time-to-cystectomy independently from other clinico-pathological factors. Given that the muscle invasive status is significantly related to BCa recurrence, VI-RADS that well reflect the imaging difference between NMIBC and MIBC, may have potential in recurrence risk stratification of BCa patients.

In addition, concerning that many surgical subspecialties, including urology, have suspended elective services and delayed many time-sensitive surgeries during the midst of COVID-19 pandemic, BCa staging is considered a priority because of the potential aggressive behavior of this disease (137). VI-RADS at the present time period may help urologist to dramatically minimize elective procedures and realize an accurate evaluation of tumor staging from a single examination, providing a prognostic criterion for adjusting oncologic class priority among overwhelmed waiting lists (137).

### Integrating the “Shallow” Features With the “Deep” Features for BCa Phenotype Diagnosis

Currently, the radiomics features adopted mainly involve the morphological features describing the geometric properties of the target region and texture features depicting the global, local and regional intensity distribution patterns of the target region (74, 115), which are designed based on certain physical or mathematical theories of the pixel intensity distribution characterized on the original images and thus can be regarded as manual or “*shallow*” features. In recent years, the radiomics features extracted by using CNN-based deep learning networks have been increasingly used to characterize the deep properties of tumors for cancer diagnosis (126, 138, 139). Owing to the black-box nature of CNN networks, the “*deep*” feature selected and the model developed seem hard to explain, limiting their applications in clinics. With the improvements in the interpretability of deep features, it is expected that the integration of shallow and deep features would provide a more precise preoperative diagnosis of the BCa phenotype.

### Macro-meso-micro Multiomics Information Fusion for More Precise, Explainable BCa Recurrence Prediction

Although both the EORTC and CUETO risk tables are extensively used as the clinical reference for NMIBC recurrence risk stratification (10), their predictive performance is far less than satisfactory (29, 120, 121, 140–142). Given that most of features in these two risk tables are macroscopic clinical factors, they may not well describe the hidden properties of BCa that are closely related to recurrence. Until now, only one study (61) has reproted the feasibility and performance of the radiomics strategy for BCa recurrence risk prediction, in which manually extracted or shallow features from a mesoscopic view were adopted in the framework.

It is now appreciated that bladder tumors are heterogeneous at the metabolomics and genomics levels (5). For example, the specific proteins and RNAs of exosomes in urine can be used as noninvasive biomarkers for BCa screening and phenotype prediction (143–149). Low-grade carcinomas can be characterized at the molecular level by loss of heterozygosity (LOH) of chromosome 9 and activating mutations of genes encoding fibroblast growth factor receptor 3 (FGFR3) and telomerase reverse transcriptase (TERT), while MIBC is thought to arise *via* flat dysplasia and Tis (5). The human epidermal growth factor receptor-2 (HER2) has been reported with overexpression among aggressive BCa for the past decade, suggesting that this biomarker might aid in patient risk stratification and treatent selection (150, 151). Ferro et al. reported that absolute basophil count is closely related to time to recurrence among patients with high-grade T1 BCa receiving BCG after TURBT (152). Whether these biomarkers can be used for BCa recurrence prediction, remains unknown. Therefore, in the future, it is believed that with macro-meso-micro information fusion of the multiomics features and multidisciplinary knowledge, the predictive performance of the recurrence risk will be greatly improved.

## Conclusion

Noninvasive imaging technologies, such as CT, contrast-enhanced CT and multiparametric MRI, and radiomic strategies can promote the overall performance of the phenotype diagnosis and recurrence risk prediction for patients with bladder cancer.

## Author Contributions

XX and HW collected and reviewed the literature. XX and HW wrote the manuscript. XX, HL, and YL helped with the writing design and revised the manuscript. YG, XZ, BL, and PD provided insightful comments and suggestions on the manuscript. All authors contributed to the article and approved the submitted version.

## Funding

This work was partially supported by the National Natural Science Foundation of China under grant (No. 81901698, 81871424, 61976248, and 82071989), Military Science and Technology Foundation under grant No. BLB19J0101, and Young Eagle Plan of High Ambition Project under grant No. 2020CYJHXXP.

## Conflict of Interest

The authors declare that the research was conducted in the absence of any commercial or financial relationships that could be construed as a potential conflict of interest.
